# Optimization of the Operant Silent Gap-in-Noise Detection Paradigm in Humans

**DOI:** 10.31083/j.jin2310183

**Published:** 2024-09-29

**Authors:** Louis Negri, Patrick Oliver, Rebecca Mitchell, Lavanya Sinha, Jacob Kearney, Dominic Saad, Fernando R Nodal, Victoria M Bajo

**Affiliations:** 1 Department of Physiology, Anatomy and Genetics (DPAG), University of Oxford, OX1 3PT Oxford, UK

**Keywords:** subjective tinnitus, humans, young adults, no hearing loss, operant behavior, auditory system

## Abstract

**Background:**

In the auditory domain, temporal resolution is the ability to respond to rapid changes in the envelope of a sound over time. Silent gap-in-noise detection tests assess temporal resolution. Whether temporal resolution is impaired in tinnitus and whether those tests are useful for identifying the condition is still debated. We have revisited these questions by assessing the silent gap-in-noise detection performance of human participants.

**Methods:**

Participants were seventy-one young adults with normal hearing, separated into preliminary, tinnitus and matched-control groups. A preliminary group (n = 18) was used to optimise the silent gap-in-noise detection two-alternative forced-choice paradigm by examining the effect of the position and the salience of the gap. Temporal resolution was tested in case-control observational study of tinnitus (n = 20) and matched-control (n = 33) groups using the previously optimized silent gap-in-noise behavioral paradigm. These two groups were also tested using silent gap prepulse inhibition of the auditory startle reflex (GPIAS) and Auditory Brain Responses (ABRs).

**Results:**

In the preliminary group, reducing the predictability and saliency of the silent gap increased detection thresholds and reduced gap detection sensitivity (slope of the psychometric function). In the case-control study, tinnitus participants had higher gap detection thresholds than controls for narrowband noise stimuli centred at 2 and 8 kHz, with no differences in GPIAS or ABRs. In addition, ABR data showed latency differences across the different tinnitus subgroups stratified by subject severity.

**Conclusions:**

Operant silent gap-in-noise detection is impaired in tinnitus when the paradigm is optimized to reduce the predictability and saliency of the silent gap and to avoid the ceiling effect. Our behavioral paradigm can distinguish tinnitus and control groups suggesting that temporal resolution is impaired in tinnitus. However, in young adults with normal hearing, the paradigm is unable to objectively identify tinnitus at the individual level. The GPIAS paradigm was unable to differentiate the tinnitus and control groups, suggesting that operant, as opposed to reflexive, silent gap-in-noise detection is a more sensitive measure for objectively identifying tinnitus.

## 1 Introduction

Subjective tinnitus is the conscious perception of a sound in the absence of any external or internal auditory stimulus, usually perceived as a ringing, humming, hissing or whistling sensation. With a prevalence of ~15% in the total population, 10% experiencing debilitating effects [1–3] and the absence of a cure, tinnitus poses a significant challenge for clinical and scientific communities.

Tinnitus is highly associated with hearing loss even if the hearing loss is not recognised by standard audiometry (hidden hearing loss) [4] and it has been suggested that some peripheral damage, for example, cochlear synaptopathy, is present in tinnitus and may be important in tinnitus generation [5,6]. In animal models of tinnitus, hidden hearing loss has been demonstrated in mice by Liberman [7] and cochlear synaptopathy has been shown in Mongolian gerbils with tinnitus induced by noise trauma [8].

Tinnitus severity in humans is commonly assessed through questionnaires, e.g., the tinnitus functional index (TFI) [9] and the tinnitus handicap inventory (THI) [10]. These two validated questionnaires assess the effects of tinnitus on daily living, scaling its severity and its negative impact on quality of life respectively. As such, TFI scores ≥17 (max out of 100) indicate a “bothersome tinnitus” phenotype [11]. Nonetheless, tinnitus is a symptom, not a condition, and recent advances in the field suggest that tinnitus as a symptom and tinnitus disorder can be differentiated by the latter being associated with emotional distress, cognitive dysfunction and/or autonomic arousal [12].

Auditory temporal resolution is essential for accurate speech perception. Silent gap-in-noise detection tests assess temporal resolution, which can be impaired by aging, neurological dysfunction, inattention, or fatigue. Whether temporal resolution is impaired in tinnitus and whether silent gap-in-noise detection tests are useful for identifying the condition is still debated. Whilst in animal models both reflexive [13–16] and operant [17–19] tests are used to identify tinnitus-like behavior, conclusions from human studies have been more tentative. For example, using the reflexive gap prepulse inhibition of the auditory startle reflex (GPIAS), Fournier and Hébert [20] showed non-frequency specific impairment in tinnitus participants whilst Campolo *et al*. [21] showed changes in the auditory startle reflex but not in GPIAS using a constant silent gap of 50 ms. On the other hand, Boyen *et al*. [22], using an operant three alternative forced choice paradigm, found no difference in performance between tinnitus participants and matched controls.

**Fig. 1:**
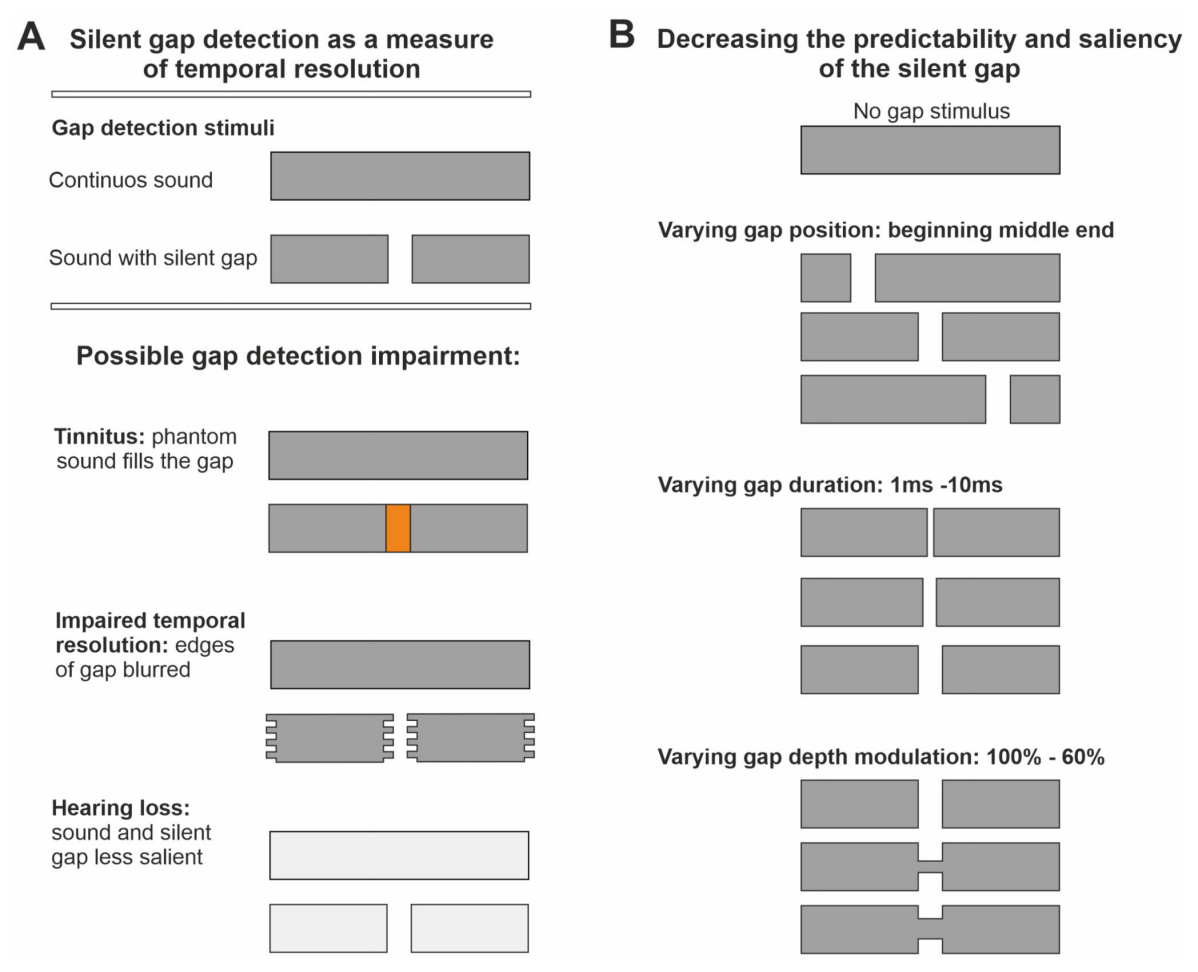
Silent gap in noise detection as a measure of temporal resolution. (A) Possible mechanisms of impairment in silent gap detection produced by tinnitus. This could be caused by the tinnitus phantom sound percept masking the silent gap, impaired temporal resolution causing the edges of the gap to become blurred, and/or tinnitus-associated hearing loss resulting in the silent gap being less salient compared to the continuous sound. (B) Diagram showing changes in gap position, duration, and depth modulation to decrease the predictability and the saliency of the salient gap. Stimuli used were 30 kHz low pass filtered broadband noise (BBN), and one octave wide narrowband noises (NBN) either centred at 1, 2, 4, 8 or 16 kHz (400 ms duration, 75 dB sound pressure level (SPL). See Materials and Methods).

We have revisited this debate in humans using an operant silent gap-in-noise two alternative forced choice detection task in combination with a reflexive behavior (GPIAS) and auditory brainstem recordings (ABRs). Our aim was to establish whether temporal resolution is impaired in tinnitus (Fig. 1A) and whether an operant silent gap-in-noise detection test is useful for identifying the condition. We tested young adults with no hearing loss or health comorbidities (other than tinnitus). We firstly optimized the operant silent gap-in-noise paradigm in 18 healthy participants without tinnitus by reducing the predictability and salience of the silent gap to avoid potential ceiling effects (where all individuals score close to optimal performance) (Fig. 1B). Our results show that operant silent gap-in-noise detection is impaired in individuals with tinnitus, suggesting impaired temporal resolution, when compared to the control group. However, in young adults with normal hearing, the paradigm is unable to objectively identify tinnitus at the individual level.

## 2 Materials and Methods

All subjects gave their informed consent for inclusion before they participated in the study. Written informed consent was obtained from each participant following the guidelines of the Ethics Committee at the University of Oxford. The study was conducted in accordance with the Declaration of Helsinki, and the protocol was approved by the Medical Sciences Interdivisional Research Ethics Committee at the University of Oxford (approval number: R57971).

The study complied with the Data Protection Act, which requires data to be anonymized. We ensured that the participants’ anonymity is maintained. The participants were identified only by a participant ID number on all study documents and computer databases.

Participants were sent an invitation letter and a participant information sheet one week before testing, to allow them to discuss their participation with third parties (e.g., General Practitioners). Participants were encouraged to read the participant information document and consent form, as well as complete questionnaires, before coming for testing. On arrival, they were briefed again and given the opportunity to ask any questions about the study before signing the consent form.

### 2.1 Study Design

**Fig. 2:**
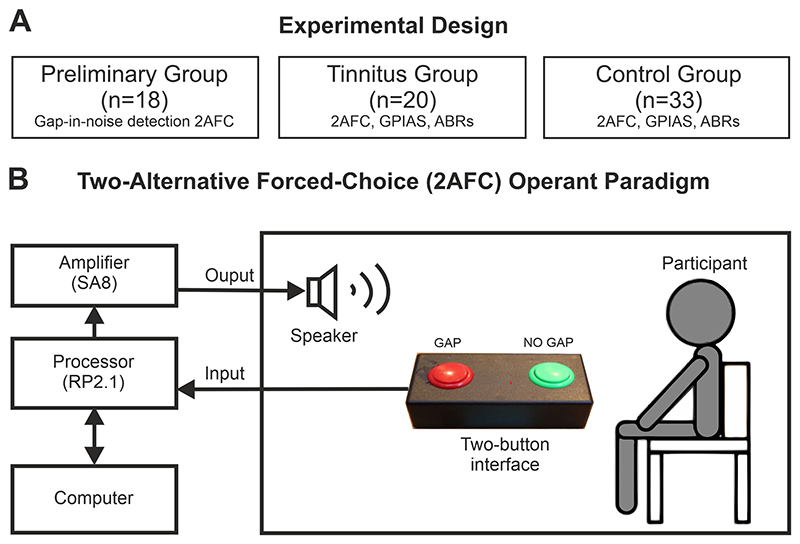
Experimental design. (A) The observational study comprised three groups: a preliminary group, where predictability and saliency of the silent gap were modified, and a tinnitus and matched-control groups, where the silent gap-in-noise detection paradigm, GPIAS, and ABRs were compared. (B) The operant behavior task was a two-alternative forced-choice (2AFC) operant paradigm with two identical sound stimuli with or without a silent gap on it. Abreviations: GPIAS, gap prepulse inhibition of the auditory startle reflex; ABRs, auditory brainstem recordings.

Seventy-one young adults participated in this study and were split into three groups: a preliminary group of healthy participants without tinnitus to optimize the silent-in-noise detection task, a tinnitus group, and a matched control group (Fig. 2A). Male and female participants between 18–30 years of age, in good health other than tinnitus were recruited from posts on the University of Oxford and Oxford Experiment Group on Facebook and invited to participate in the study. Inclusion criteria were age (18–30 years), normal or corrected-to-normal vision, no diagnosed health conditions other than tinnitus, and availability for 12 months after starting the study. Exclusion criteria were any major medical or psychiatric condition (other than tinnitus), hearing loss (defined as >25 dB hearing level (HL) in both ears), severe hyperacusis (assessed using the Khalfa hyperacusis questionnaire [23,24]), claustrophobia, or belonging to a vulnerable group.

A general questionnaire was used to assess age, gender, current educational status, current educational area, previous education, free school dinner eligibility, languages spoken in order of familiarity, highest music grade and the instrument on which it was obtained, whether participants were claustrophobic or dyslexic, history of hearing problems, medical conditions and any current medications. Tinnitus sufferers answered additional questions which assessed their history of tinnitus, duration of chronic tinnitus, whether the tinnitus is pulsatile and previous or ongoing tinnitus therapy.

The Edinburgh Handedness Inventory (EHI; [25]) was used to assess the participant’s predominant hand and the Khalfa Hyperacusis Questionnaire (HQ; [23,24]) was used to assess hyperacusis. Tinnitus severity was assessed by using the Tinnitus Functional Index (TFI; [9]) and Tinnitus Handicap Inventory (THI; [10]). In addition, if individuals with tinnitus perceived their tinnitus as a hearing disability, we assessed the significance of any hearing disability using the Hearing Handicap Inventory (HHI; [26]).

The first group of eighteen individuals without tinnitus (preliminary group) was allocated to examine the effect of the position and saliency of the silent gap within the stimulus sound on the performance of the operant silent gap in-noise detection paradigm (preliminary experiment). The other participants (n = 53) were a tinnitus group (n = 20) and a matched control group (n = 33) used in a case control observational study. Tinnitus participants were accepted if they had chronic tinnitus (>3 months) that was not pulsatile (following a heartbeat rhythm), not objective (noise that can be heard by an external examiner or created internally in the body) or a consequence of a medical disorder. Tinnitus participants were not accepted if they were undergoing any tinnitus therapy at the same time as our study.

In the preliminary group, only operant silent gap-in-noise detection was tested following questionnaires and audiogram. The optimized silent gap-in-noise detection paradigm was then tested in the tinnitus and control groups, alongside silent GPIAS and ABRs (Fig. 2A).

The tests were organized into blocks of 100 trials each, where the duration and position of gaps were randomized whilst the type of sounds (30 kHz low pass filtered broadband noise (BBN), and one octave narrowband noises (NBN) either centred at 1, 2, 4, 8 or 16 kHz) and gap amplitude modulations (100%, 80%, 60%) were kept fixed in each block. To maintain subject focus and attention, testing was spread over several one hour-long sessions with regular breaks. It took between 3 and 5 hours to complete the testing. This was predominantly carried out in a single day for each participant, although on occasions was performed over two days when necessary due to tiredness. All testing was conducted in a double-walled sound-attenuating chamber (IAC acoustics, Winchester, UK; dimensions 1020 wide, 1250 deep and 2000 mm high).

Audiometry testing followed the standard audiometry guidelines (2018) from the British Society of Audiology [27], updated to include testing at 16 kHz. Pure tone bursts (0.25, 0.5, 1, 2, 4, 6, 8, 16 kHz) at different intensity levels (generated using an RP2.1 real-time processor, 100 kHz sampling rate) and attenuated using a PA5 programable attenuator (TDT, Alachua, FL, USA) were presented to each ear separately through headphones (HD 250, Sennheiser, Wedemark, Germany) to extract hearing thresholds. Any frequency threshold above 25 dB transformed HL resulted in exclusion from the study. Two participants from the control group failed to meet this criterion. Tinnitus participants self-matched their tinnitus percept at each ear to a computer-generated pure tone presented over headphones by varying the frequency (0.25 to 16 kHz) and intensity of the pure tone (0–90 dB sound pressure level, SPL). One participant reported hearing a “white noise” tinnitus and was excluded from the study. Another participant with pulsatile tinnitus was also excluded.

### 2.2 Operant Silent Gap-in-Noise Detection

Participants were exposed to sound stimuli (400 ms duration, 75 dB SPL) from a speaker (TW026MO, Audax, AAC, La Chartre Sur le Loir, France) positioned level with their heads. The stimuli used were 30 kHz low pass filtered BBN, and one octave NBN either centred at 1, 2, 4, 8 or 16 kHz (computed in MATLAB v2022, MathWorks, Natick, MA, USA) and generated using a real-time processor RP2.1 (TDT, Alachua, FL, USA), sampling rate 100 kHz, and amplified by an SA8 power amplifier (TDT, Alachua, FL, USA). In half of the trials, a silent gap was inserted pseudo-randomly, either at 100, 200 or 300 ms from the start. Using a two-alternative forced-choice paradigm, participants pressed the left button to indicate that they had heard a silent gap and the right button to indicate that they heard a continuous sound (Fig. 2B). The task events, stimulus presentation and responses were controlled by custom scripts written in MATLAB controlling the TDT hardware (RP2.1 and SA8).

Testing was performed in blocks of 100 trials where the silent gap duration was randomized whilst the type of stimulus was fixed (BBN or 5 different NBN one octave narrowband noises either centred at 1, 2, 4, 8 or 16 kHz). Six independent blocks of 100 trials each were performed by each participant. In the preliminary group, the depth of the gap was modulated at 100%, 80% and 60% intensity attenuation (named mod 0 at 100%, mod 2 at 80% and mod 4 at 60% intensity modulation). This was repeated in independent testing blocks for each stimulus type and gap modulation combination (18 blocks in total: 6 types of stimulus and 3 gap depth modulations). For each of the 18 combinations, the duration of the silent gap was varied at either 1, 3, 4, or 10 ms between trials.

In the control case observational study, both control and tinnitus groups were presented with a wider range of silent gaps (1, 3, 5, 10, 20, 50, 100 and 270 ms), the position of the silent gaps in each stimulus was randomly varied in each trial and 80% modulation depth was used for all gap trials. Blocks of 100 trials were used for each stimulus type combination (6 blocks in total).

For each participant, eleven supervised training trials were conducted before data acquisition to allow familiarization with the equipment and sounds presented. To prevent a testing learning effect over time, the order of frequencies and modulations tested was randomized [28,29] in each participant from the three experimental groups. To guide them during testing, a monitor displayed the trial number and a reminder of what each button meant: the left display box read “two noises” and the right display box read “one noise” (Fig. 2B). These labels were used instead of “gap” and “no-gap” to reduce the cognitive load.

### 2.3 Silent Gap Prepulse Inhibition of the Acoustic Startle Reflex (GPIAS)

The eye blink reflex (Preyer reflex) in response to a startle stimulus was measured using the surface electromyographic signal (sEMG) of the left orbicularis oculi muscle [30]. Surface electrodes (AG/AgCl non-metallic radiolucent 10 mm diameter disposable EEG disc electrodes, filled with Ten20 conductive Neurodiagnostic Electrode Paste, Weaver & Co, Aurora, CO, USA) were placed on the right side of the face and attached to the skin using skin-safe tape. To minimize movement artefacts and standardize attention across participants, participants were invited to play ‘Where’s Wally?’ during testing. Electrical signals were amplified and digitised (25 kHz) by a low impedance headstage (RA4LI-RA16PA) connected to a RA16 and controlled by Synapse software (v.95, TDT, Alachua, FL, USA).

Participants were presented with 40 startle stimuli, 100 ms BBN 120 dB SPL, on a background of continuous 60 dB SPL BBN [31]. These parameters typically elicit a blink response [32], limit habituation [33], and minimize boredom or discomfort. In half of the trials, a 50 ms silent gap was introduced within the background noise, 150 ms before the startle stimulus. These gaps were incorporated pseudo-randomly. The interval between each trial was also pseudo-randomly allocated between 6–17 s to reduce predictability [31–34].

We used the logarithmic acoustic startle reflex ratio as a proxy measure of pre-pulse inhibition (PPI, following Ornitz [35]).

$$\text{PPI}\;=\;1-\left(\frac{\text{Startle}\;\text{Amplitude}\;\text{in}\;a\;\text{gap}\;\text{trial}}{\text{Startle}\;\text{Amplitude}\;\text{in}\;a\;\text{no-gap}\;\text{trial}}\right)$$

Using rodent models of tinnitus, Schilling *et al*. (2017) [34] demonstrated a statistical approach to measuring PPI. They created a vector of all possible combinations of gap and no-gap trial amplitude ratios (ASR). Through trialling several transformations, they found logarithmic ASR, log(ASR), to be Gaussian-like distributed. We bootstrapped log(ASR) for each individual with 2000 re-samples to obtain a distribution of log(ASR) means. We used the mean of this distribution as the proxy measure for participant pre-pulse inhibition.

To exclude unrelated blinking, we only considered EMG responses which occurred 21–120 ms after stimulus presentation [31] and excluded responses with an amplitude lower than the mean of the baseline plus 2 standard deviations. Response latency was taken as the time between stimulus onset and maximum EMG amplitude within the response window.

### 2.4 Auditory Brainstem Responses

Using headphones, participants were presented with clicks of alternating polarity at a rate of 89.9 Hz and pure tones (2 and 4 kHz). Stimuli were presented 2000 times in 4 blocks initially of 0, 15, 30, and 45 dB HL. However, due to difficulties in isolating individual peaks, the HLs were later increased to 75, 80, 85, and 90 dB HL. The stimulus presentation rate was 27.7 Hz. Sentiero equipment and software (v2.5.1, Path Medical, Germering, Germany) were used to produce the stimuli. ABRs were recorded using surface electrodes (DORMO SX-30, Ag/AgCl E.C.G./E.K.G., Telic Group, Bigues i Riells, Spain) with Signa gel (Parker Laboratories, Fairfield, NJ, USA), a highly conductive multi-purpose electrolyte electrode gel if the impedance was lower than 6 kΩ. Electrodes were placed on the right side of the head.

### 2.5 Data Analysis and Statistical Treatment

Data analysis was performed with MATLAB (Math-Works), and R (version 4.3, R Foundation for Statistical Computing, Vienna, Austria). All data were analysed using custom-written scripts in MATLAB. We tested for normality (Shapiro Wilk [SW] test when n <50 and Kolmogorov-Smirnov [nKS] test when n >50) and according to the outcome we either used parametric (Levene’s Test for homoscedasticity followed by analysis of variance (ANOVA) and post-hoc Bonferroni test) or non-parametric tests (Kruskal-Wallis [KW] test followed by either Mann-Whitney U [MWU] or Kolmogorov-Smirnov [KS] tests) for group comparisons. *χ*^2^ tests of proportions were used to assess minimal clinically important difference in TFI scores, and Pearson correlation coefficients were used to relate changes in TFI scores to changes in tinnitus duration. Two-sided *p <* 0.05 indicated statistical significance. ABRs peak amplitude and latency values extracted by Sentiero software were exported for analysis in MATLAB.

**Fig. 3:**
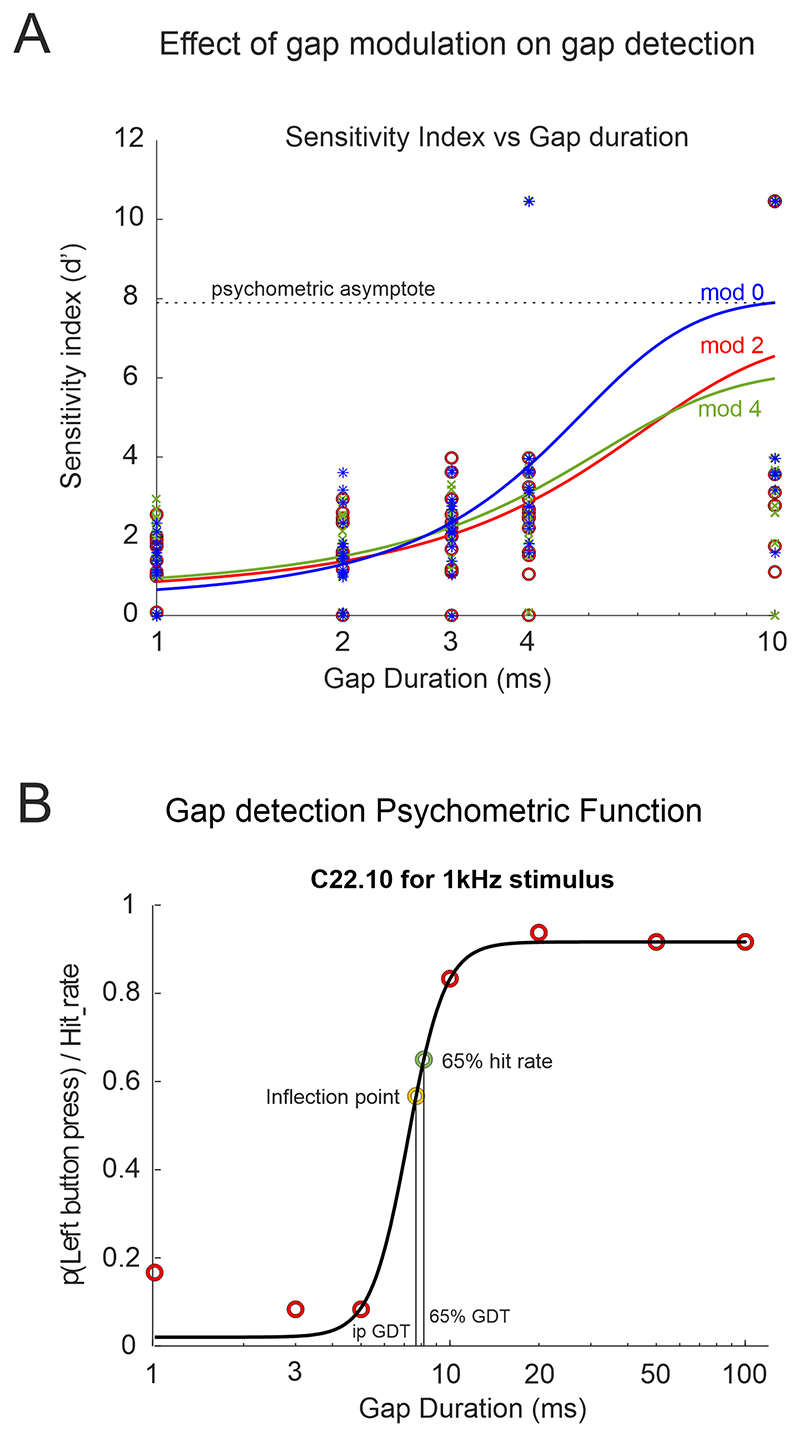
Examples of gap detection psychometric functions. (A) Silent gap durations against the sensitivity index (*d’*) for the preliminary experiment. (B) Psychometric curve plotting left button press probability against gap duration for the case control observational study (control participant C22.10), equivalent to the hit rate for silent gap trials and the false alarm rate for no gap trials. Abbreviations: ipGDT, gap detection threshold corresponding to the inflection point; 65% GDT, gap detection threshold defined at 65% probability; mod 0, 2, 4 gap modulation depth 100, 80, 60% respectively.

For operant silent gap-in-noise detection in the preliminary experiment the sensitivity index, *d’*, was calculated to adjust the hit rate (HIT) for false alarms (FA):

$$d^{\prime }=z(HIT)-z(FA)$$

where *z(x)* is the inverse normal distribution z-score of the proportion *x* (HIT or FA). Values of *d’* were adjusted when HIT was equal to 1 or FA was equal to 0; in these instances, *d’* was estimated as being 1–(1/2*T*) or 1/2*L*, respectively, where T is the number of targets and L is the number of lures. Subject accuracy was plotted against gap length to produce psychometric function curves. Gap detection threshold was defined as the gap length when *d’* = 1, corresponding to approximately 60% correct responses in the task. The slope of the psychometric function at threshold (Fig. 3A) was also used as a measure of sensitivity. Optimal performance was defined by the maximum value of *d’* and the lapse rate (the proportion of incorrect responses at the longest gap length (10 ms), for which high values may indicate a lack of attention). The statistical significance of the results was tested using multilevel ANOVA with subject identity (ID) as a random factor. Results are reported as mean ± standard error (SE). To assess the statistical significance in gap detection thresholds and slopes, we used a bootstrap procedure (200 repeats) in which *d’* values were calculated after resampling the trials to produce a range of psychometric functions for each stimulus type, which formed a reference distribution. From this distribution, we then performed statistical analysis using multilevel ANOVA.

In the observational study that included control and tinnitus participants, due to adding longer gap durations (>10 ms, maximum 270 ms), fitting of the psychometric function using *d’* was less reliable than using the left button press probability. Therefore, the latter metric was used to generate the psychometric curves (Fig. 3B), to which we fitted sigmoid functions. This is equivalent in signal detection theory to plotting the hit rate for silent gap trials and the false alarm rate for no gap trials (example shown in Fig. 3). Performance was assessed by the gap detection thresholds (GDT) defined as (1) the gap duration at which the participant’s hit-rate was 65% (65% GDT) and (2) the gap duration at the psychometric inflection point (the point on a curve where the gradient changes sign - ipGDT), both as estimators of their gap detection threshold; (3) the maximum asymptote as an indication of maximum performance; (4) the slope gradient to estimate sensitivity; (5) and the false alarm rate for no gap trials to quantify attention/bias.

Performance of the silent gap-in-noise detection paradigm for durations greater than 100 ms was poorer than predicted. This could be explained by both inattention and pre-emptive button pressing. Therefore, data for gap-in-noise of durations greater than 100 ms were only included if the performance exceeded those for silent gap-in-noise durations of 50 ms and less. Participant data was also excluded if their FA rate was over 50%, indicating inattention and/or bias towards detecting silent gaps.

## 3 Results

All participants in the three groups (preliminary, tinnitus and control) were young adults well-matched in terms of age, sex ratio, educational status, socioeconomic group, language ability and handedness. Initially, left-handed participants were excluded from the analysis due to the potential for reversed lateralization and a consequent increase in variance, reducing statistical sensitivity. No differences were found in the silent gap-in-noise detection response times based on predominant hand, so we decided to include both right and left-handed participants.

### 3.1 Preliminary Group: Decreasing the Saliency and Predictability of the Silent Gap Makes it Harder to Detect

**Fig. 4:**
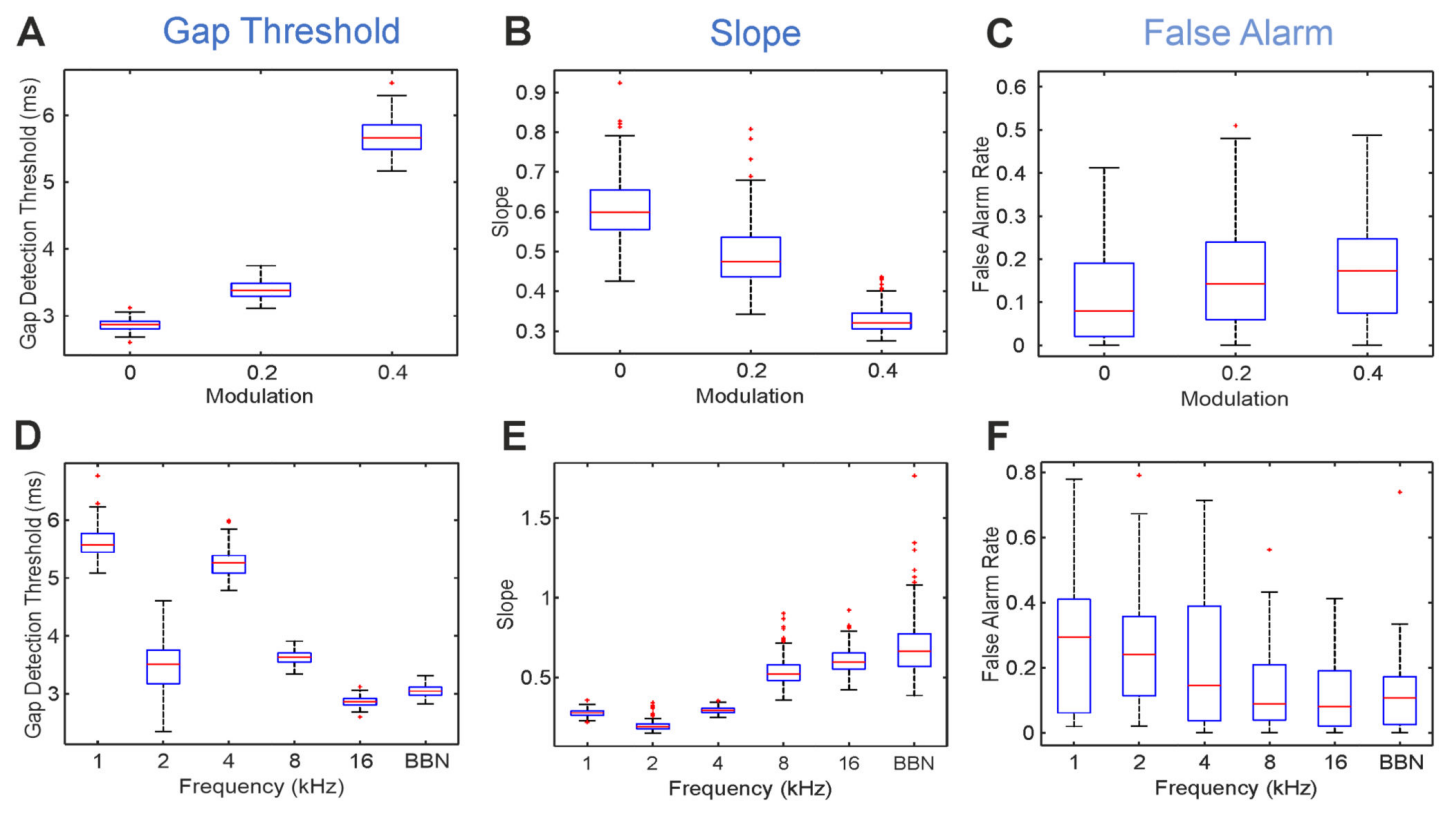
Variation of gap position, gap intensity modulation and sound stimulus type changes the gap detection threshold, slope and false alarm. Changes in threshold (A), slope (B) and False Alarm rate (C) produced by varying the gap intensity modulation for 16 kHz narrowband noises (NBN) are depicted. Changes across stimulus types (different spectral composition) without modulation (mod = 0) on threshold (D), slope (E) and False Alarm rate (F). 1–16 centre frequency in kHz for 1-octave wide narrowband noises. Abbreviations: BBN, broadband noise.

In the preliminary group experiment (n = 18), both gap position (100, 200, 300 ms) and attenuation of the sound intensity during the gap (depth modulation 100% (mod 0), 80% (mod 2) and 60% (mod 4)) were varied randomly between individual trials with the aim of decreasing saliency and predictability (Fig. 1B). Salience was found to depend on depth modulation, with salience increasing with depth modulation. Silent gap detection thresholds increased when salience and predictability of the gap were reduced (Fig. 3A and Fig. 4). No differences were found in the reaction time (ranging between 800 and 900 ms) regardless of the modulation depth used, the position of the gap, or the type of stimulus (BBN or one octave NBN centred at 1, 2, 4, 8, 16 kHz).

Due to limited data, we used hit and lapse rates, rather than *d’* prime, to estimate performance according to gap position (Fig. 3A, Table 1). We found that for gap lengths ≥3 ms, hit rate was significantly higher when the gap was in the middle compared to the beginning of the stimulus (Table 1). *Post-hoc* comparison revealed that gaps at the end of the stimulus had the lowest hit rates (mixed-effects ANOVA, *p <* 0.01). However, the position of the gap had no effect on hit rates (proportion of correct responses) when the gap was as short as 1–2 ms, suggesting that this is close to the limit of detection.

Lapse rates followed the same pattern as hit rates according to performance. For all stimulus types, gaps in the middle of the stimulus produced the lowest lapse rates, gaps at the beginning produced significantly higher lapse rates and gaps at the end produced the highest lapse rates (mixed-effects ANOVA, *p <* 0.01; Table 1).

The reduction of gap salience through depth modulation had a significant effect on its detection indicated by the rising detection thresholds (Fig. 4A), decreased sensitivity (Fig. 4B) and increased false alarm rates (Fig. 4C). When assessing each stimulus type, gap detection thresholds were significantly higher at increased modulation values for all narrowband sounds except 8 kHz. Gap detection threshold at 4 kHz NBN, for example, increased from 5.23 ± 0.05 ms with no modulation to 6.86 ± 0.17 ms at 80% intensity modulation and further to 8.92 ± 0.04 at 60% intensity modulation (*F*_(2,5)_ = 183.55, *p <* 0.001). The change in thresholds observed at higher modulations was greatest for 2 kHz NBN stimuli, for which threshold was 3.46 ± 0.10 ms with no modulation, 5.56 ± 0.22 ms at mod 2 modulation, and 9.23 0.31 ms at mod 4 (*F*_(2,5)_ = 725.15, *p <* 0.001). Without modulation, the gap detection threshold at 2 kHz NBN is more similar to the detection thresholds of the higher frequencies (8 kHz and 16 kHz) and BBN than the lower frequencies (Fig. 4D. *F*_(2,5)_ = 4685.1, *p <* 0.001). The fact that 2 kHz NBN has a particularly low gap detection threshold at low modulation, but the highest at high modulation, suggests that as a frequency it is the most sensitive to the effects of modulation of the silent gap.

The slope of the psychometric function at *d’* = 1 (threshold) indicates the dynamic sensitivity of the test, with higher slopes associated with a greater increase in *d’* to small gap length differences (Fig. 3A). Slope depends on the spectral quality of the stimulus. As with gap detection threshold, lower frequencies (1 kHz; 2 kHz and 4 kHz NBN) were associated with flatter slopes, and higher frequencies (8 kHz, 16 kHz NBN and BBN) with steeper slopes (at *d’* = 1. *F*_(5,199)_ = 495.29, *p <* 0.001. Fig. 4E). This effect suggests that silent gaps are easier to detect in stimuli containing higher frequencies. This manifests as lower gap detection thresholds (Fig. 4D), steeper slopes (Fig. 4E) and reduced variability of false alarm rate (Fig. 4F) at higher frequencies.

Gap detection sensitivity (slope) also depends on the modulation of gap stimuli (Fig. 4B). For stimuli with higher modulation, slopes were less steep (*F*_(2,199)_ = 205.03, *p <* 0.001) (slope at *d’* = 1 [fraction], mean ± SE: mod 0: 0.44 ± 0.02; mod 2: 0.40 ± 0.01; mod 4: 0.34 ± 0.01). Where gap detection threshold values were very similar for different modulations (e.g., at 8 kHz NBN and BBN stimuli, where the range of gap detection thresholds across all modulations was as low as 0.28 ms), slope provides some discrimination in subject performance.

False alarm rates varied across individual subjects according to their internal decision criterion value. However, if subject identity is nested as a random factor, inter-individual variability can be accounted for, and differences in FA across conditions may be interpreted as a useful performance indicator. Modulation and stimulus type had a significant effect on FA (Fig. 4C,F). FA is dependent on gap modulation, with greater FA rates at higher values of modulation (FA rate [fraction], mean ± SE: mod 0: 0.19 ± 0.01; mod 2: 0.23 ± 0.02; mod 4: 0.25 ±0.02; *F*_(2,17)_= 6.01, *p <* 0.01). An increase in FA across all individuals at higher modulation values suggests that for mod 2 and mod 4 stimuli (Fig. 4B), it is progressively harder to discriminate gap and no-gap stimuli.

Higher frequency stimuli were associated with lower FA rates than lower frequency stimuli (*F*_(5,17)_ = 3.53, *p <* 0.001. Fig. 4F). This indicates that for lower frequency stimuli, subjects were more likely to guess the presence of a gap, in turn suggesting they found these harder to discriminate (Fig. 4F).

Lapse rates (the proportion of gap-containing stimuli with maximal gap length (10 ms) that the subject failed to detect) can be used to indicate subject attention, as 10 ms gaps are the easiest gaps to identify and are generally consistently above threshold values. For all stimulus types, lapse rates were higher with greater gap modulation (lapse rate [fraction], mean ± SE: mod 0: 0.10 ± 0.01; mod 2: 0.16 ± 0.02; mod 4: 0.30 ± 0.03; *F*_(2,17)_ = 4.43, *p <* 0.01).

Lapse rates at mod 4 were particularly high. Additionally, mod 4 stimuli were significantly affected by stimulus type (*F*_(5,7)_ = 5.46, *p <* 0.01) (lapse rate [fraction], mean ± SE: 1 kHz NBN: 0.42 ± 0.07; 2 kHz NBN: 0.39 ± 0.08; 4 kHz NBN: 0.46 ± 0.07; 8 kHz NBN: 0.07 ± 0.03; 16 kHz NBN: 0.30 ± 0.04; BBN: 0.19 ± 0.08). *Post-hoc* comparison revealed that the lapse rates for 1 kHz NBN, 2 kHz NBN, 4 kHz NBN and 16 kHz NBN were significantly different to 8 kHz NBN and BBN at mod 4, but that stimulus types within these latter two groups were not significantly different to one another.

Although the participants were not given any instructions regarding the timing of their responses, it is thought that the response time (RT) is indicative of the responder’s confidence, and that this will vary according to the difficulty of the task. There was a significant difference in the mean RT for some subjects (*F*_(17,405)_ = 50.14, *p <* 0.001), demonstrating the individual variability of the cohort. However, when subject ID was nested as a random factor, it revealed no significant effect of gap modulation on RT (*F*_(2,17)_ = 0.38, *p* = 0.68).

For most gap lengths, there was no difference in mean RT, but for 10 ms gap stimuli the mean RT was significantly lower than for other gap lengths (Supplementary Fig. 1A; *F*_(5,17)_ = 3.94, *p <* 0.01). This suggests that subjects recognized 10 ms gaps more rapidly than stimuli with other gap lengths (including no-gap stimuli). Stimulus type had a significant effect on RT, even when subject ID was included as a nested random factor (*F*_(5,17)_ = 4.23, *p* = 0.001). Whilst *post-hoc* comparisons were not significant between the RTs for 1 kHz, 2 kHz and 16 kHz NBN, mean RT for 8 kHz NBN stimuli was lower than the RTs for 4 kHz NBN and BBN stimuli (Supplementary Fig. 1B; *F*_(5,17)_ = 4.23, *p* = 0.001).

### 3.2 Comparisons between Tinnitus and Control Groups

Next, we discuss the results of the main study which compared a tinnitus cohort with matched-controls. Hearing status was established by audiometry and ABR measurements (see methods). Two control participants were excluded due to unilateral hearing loss and two tinnitus participants were excluded for not meeting the inclusion criteria. Between the control and tinnitus groups, there were significant differences in musical ability (Table 2) with controls having a median of 8 in their best musical grade whereas the tinnitus group had only 4 (Mann-Witney *U* Test, *U* = 755, *p* = 0.0014). Significant differences were also observed in the hyperacusis questionnaire with higher scores in the tinnitus group (Table 2). The modified Khalfa Hyperacusis questionnaire comprises of 14 items that evaluate someone’s sensitivity to everyday sounds within the attentional, social and emotional domains. The maximum score is 42 points. Tinnitus participants scored 39 versus 25 in controls (Table 2; Mann-Witney *U* Test, *U* = 684.5, *p* = 0.0018).

Following audiometry, the tinnitus percept for the tinnitus group was individually assessed in each ear. This was achieved by tinnitus participants self-matching their tinnitus percept to a computer-generated pure tone presented over headphones by varying the frequency (0.25 to 16 kHz) and intensity (0–90 dB SPL) of the pure tone (see methods). Despite matching the tinnitus of both ears independently, there was a strong intra-individual correlation between the tinnitus percept in each ear for most of the tinnitus participants (frequency *r* = 0.7528; intensity *r* = 0.6479). The median tinnitus frequency identified for tinnitus participants was 9.2 kHz for the left ear and 8.9 kHz for the right ear, with median intensity levels of 22 and 26 dB SPL respectively. Therefore, the tinnitus percept was close to the hearing threshold (19 dB for 8 kHz pure tone (ISO 389-5 part 2)) in the majority of the tinnitus cohort (Table 3).

### 3.3 Silent Gap Detection is Impaired in Tinnitus Participants Compared with Controls

**Fig. 5:**
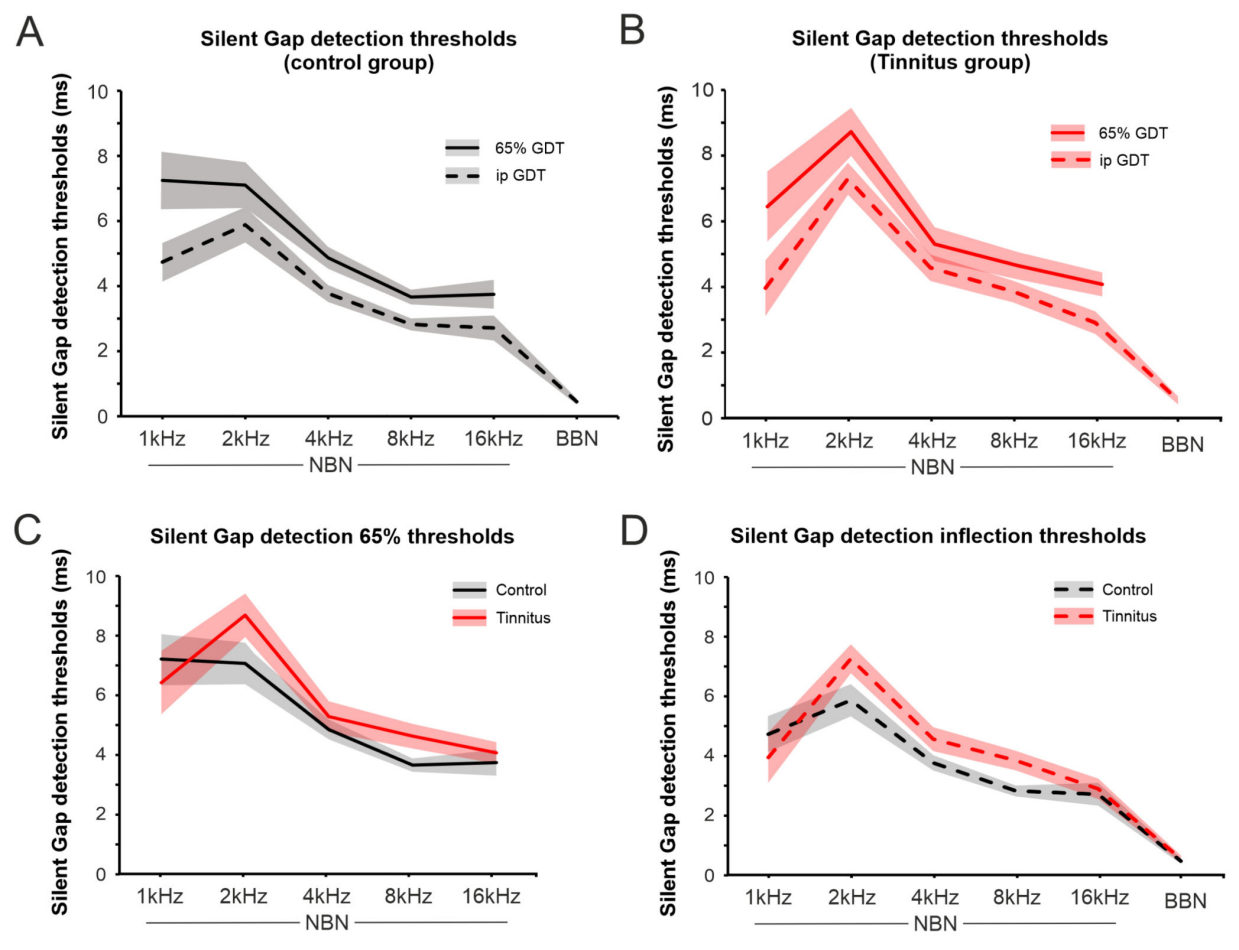
Gap detection thresholds in control and tinnitus. (A) Changes in gap detection threshold (GDT) in the control group using the 65% hit rate (65% GDT, solid line) and the inflection point (ipGDT, dashed line) values for the different type of stimulus used. The average gap detection thresholds at 65% performance. (B) Changes in gap detection threshold in the tinnitus group using the 65% hit rate (solid line) and the inflection point (dashed line) values for the different type of stimulus used. (C,D) show the comparison between control and tinnitus groups for the different stimulus used with 65% GDT (C) and ipGDT (D). The silent gap detection inflection threshold was lower overall for control than tinnitus participants (2-way ANOVA, *F*_(1,24)_ = 12.3, *p <* 0.01). *Post hoc* analysis revealed this difference was significant at 2 and 8 kHz NBN (Bonferroni test, *p <* 0.05). Solid and dashed lines represent the averages of 65% GDT and ipGDT, respectively, with the shadows representing the standard error of the mean (sem). In black the controls and in red the tinnitus group. 65% GDT for broadband noise (BBN) was not represented for controls in (A) or for controls and tinnitus in (B) because only 3 datapoints in the controls present with lower than 65% hit rates whilst all other participants had higher hit rates.

Gap detection thresholds were estimated according two criteria: when hit rate was 65% (65% GDT) and at the inflection point in the psychometric function (ipGDT) (Fig. 3B; see methods). Both estimations, 65% GDT and ipGDT, show similar trends across the stimuli used (BBN and NBN 1, 2, 4, 8, 16 kHz; Fig. 5A) and with good correlation between them (Table 4).

All participants exhibited the same relationship between the gap detection threshold and stimulus type of the sound presented. Thresholds were overall lowest for BBN stimuli. Among NBN stimuli, thresholds increased as stimulus frequency decreased, independently of the threshold estimator used, although ipGDT had a lower variance than 65% GDT (Fig. 5, Table 4).

Overall, the controls had lower gap detection thresholds than tinnitus participants (Fig. 5; 2-way ANOVA, *F*_(1,24)_= 12.3, *p <* 0.01). This trend in difference was observed for all stimulus types tested except 1 kHz NBN (the lowest frequency tested). *Post hoc* analysis revealed that the differences were statistically significant at 2 and 8 kHz NBN (Student’s *t*-test, *p <* 0.05). The differences at 2 kHz NBN for 65% GDT (6.96 *vs*. 6.20 ms; *p* = 0.032, KS) (Fig. 5C) and 8 kHz NBN for ipGDT (3.87 *vs*. 3.11 ms; *p* = 0.026, MWU) (Fig. 5D) were statistically significant.

Although the tinnitus was always chronic (>3 months), the duration of the tinnitus ranged from 5 to 120 months with a median of 44 months (Table 3). In the tinnitus group, we found that gap detection thresholds for NBN stimuli centred at and below 4 kHz were positively correlated with the duration of the tinnitus (Supplementary Fig. 2), suggesting that gap detection performance worsens with tinnitus duration.

### 3.4 Tinnitus Questionnaires (TFI, THI, and the Combined Index) Correlate with Operant Testing in Tinnitus Participants

**Fig. 6:**
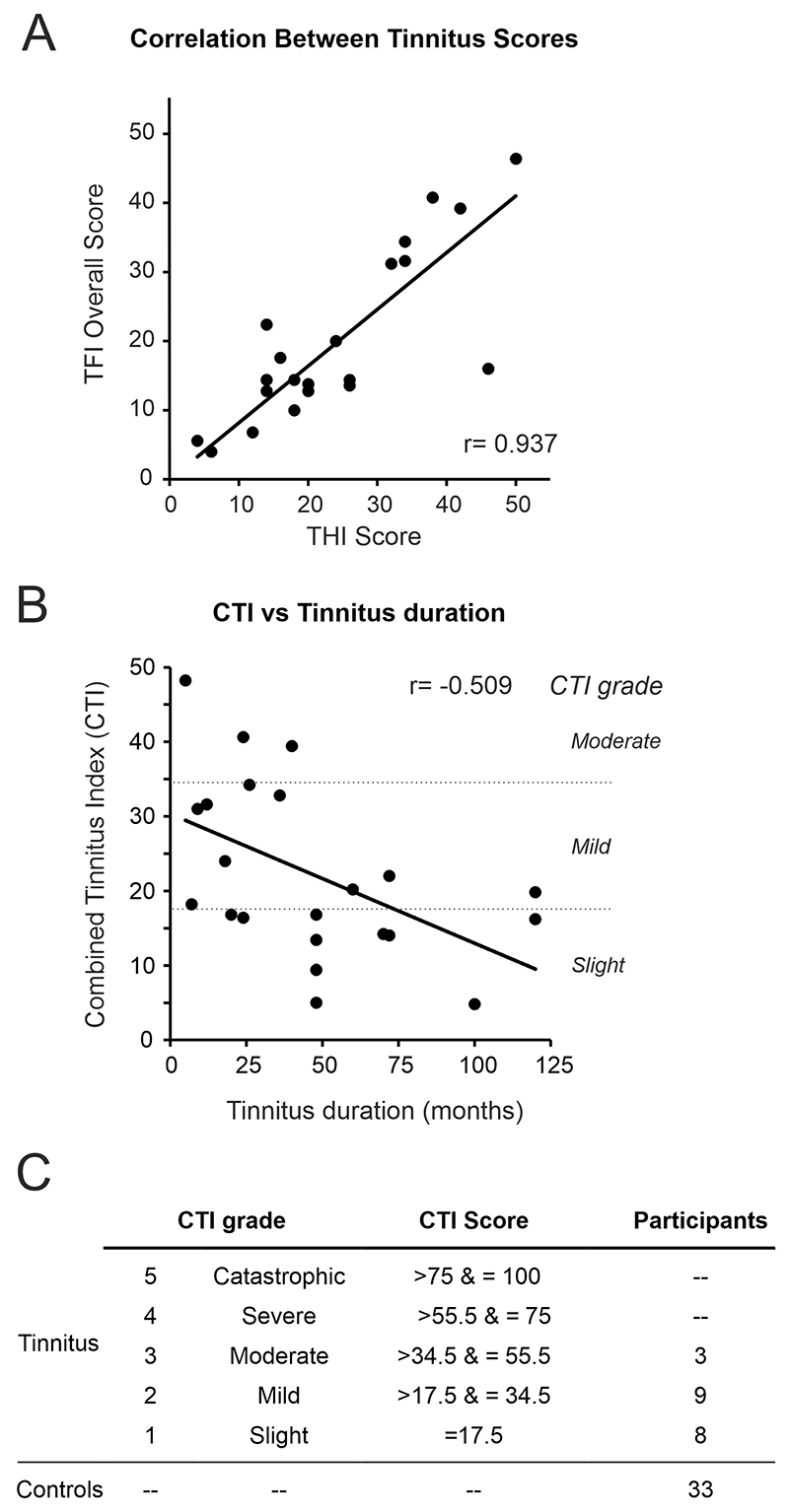
Correlation of Tinnitus questionnaires. (A) Tinnitus functional index (TFI) and tinnitus handicap inventory (THI) scores were highly correlated (*r* = 0.937) and the combined tinnitus index (CTI) was calculated. (B) CTI scores and tinnitus duration were negatively correlated (r = –0.555, *p* = 0.007). (C) Tinnitus participants were stratified into groups of tinnitus severity according to their CTI scores.

Tinnitus participants completed two different questionnaires to assess the impact of their tinnitus: The TFI, with 8 domains (intrusiveness, sense of control, cognitive, sleep, auditory, relaxation, quality of life, and emotional), and the THI (Table 5). Scores from both questionnaires were highly correlated (Pearson Correlation *r* = 0.937) (Fig. 6A), so we calculated a combined tinnitus index (CTI) as the mean of the two questionaries’ scores. CTI scores negatively correlated with the tinnitus duration (*r* = –0.555. Fig. 6B), indicating that the longer the tinnitus duration the lower the impact it had on subject’s daily life.

Using CTI scores, we categorised participants into 5 groups (Fig. 6C) following the same criteria used for TFI [9]. A CTI of 0 was given to controls for comparison purposes. We found that tinnitus participants tended to have low CTI scores (slight to moderate) (Fig. 6C; Table 5). Interestingly, all TFI subcategory scores increased with CTI scores, except for the audiological subcategory (Table 5).

**Fig. 7:**
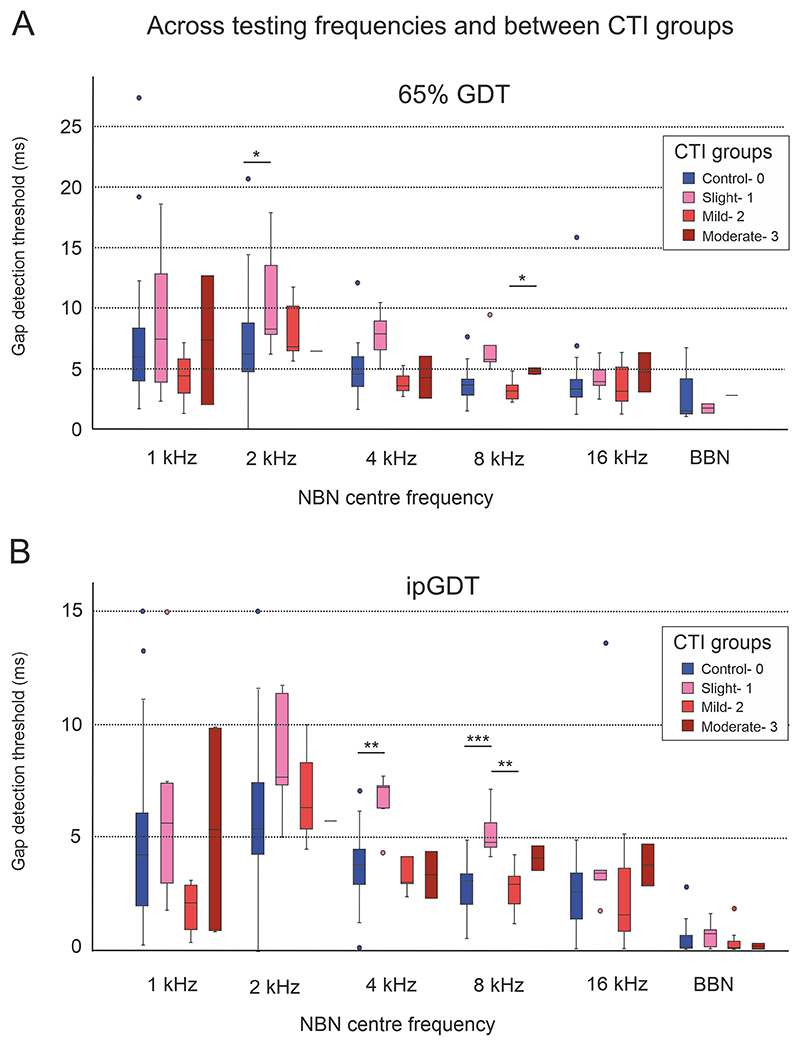
Comparing 65% GDT (A) and ipGDT (B) gap detection thresholds between controls and tinnitus cohorts stratified by CTI grades. Those with slight tinnitus (CTI 1) had elevated GDTs relative to controls, for 4 kHz (7.89 *vs*. 4.58 ms, *p* = 0.007, 65% GDT; 7.22 *vs*. 3.8 ms, *p* = 0.002, ip GDT; Mann-Whitney U (MWU)) and 8 kHz (5.73 *vs*. 3.464 ms, *p* = 0.002, 65% GDT; 4.8 *vs*. 3.11 ms, *p* = 0.001, ipGDT; MWU) stimuli and to those with mild tinnitus (CTI2), for 4 kHz (*p* = 0.007, 65% GDT; MWU) and 8 kHz (*p* = 0.004, 65% GDT; *p* = 0.007, ipGDT; MWU) stimuli. Statistical significance is indicated by asterisks, * *p <* 0.05, ** *p <* 0.01, *** *p <* 0.001.

When comparing control and tinnitus cohorts stratified by CTI grade, those with slight tinnitus (CTI 1) had greater 65% GDTs relative to controls for 4 kHz (7.89 *vs*. 4.58 ms, *p* = 0.007, 65% GDT; 7.22 *vs*. 3.8 ms, *p* = 0.002, ipGDT; MWU) and 8 kHz NBN (5.73 *vs*. 3.464 ms, *p* = 0.002, 65% GDT; 4.8 *vs*. 3.11 ms, *p* = 0.001, ipGDT; MWU). For the same frequencies, these individuals with slight CTI 1 tinnitus also had greater 65% GDTs compared to those with and relative to those mild CTI 2 tinnitus (for 4 kHz *p* = 0.007, 65% GDT; MWU and 8 kHz *p* = 0.004, 65% GDT; *p* = 0.007 ipGDT; MWU) (Fig. 7A,B).

As previously mentioned, a negative Spearman’s correlation was found between CTI score and tinnitus duration (–0.555, *p* = 0.007). Linear regression analysis revealed that 26% of the variation in CTI could be accounted for by tinnitus duration. Longer tinnitus duration might explain the gap detection threshold impairment found in CTI 1, the group where tinnitus had the lowest functional impact measured by the questionnaires.

### 3.5 Silent GPIAS did not Show Differences between Tinnitus and Control Participants

**Fig. 8:**
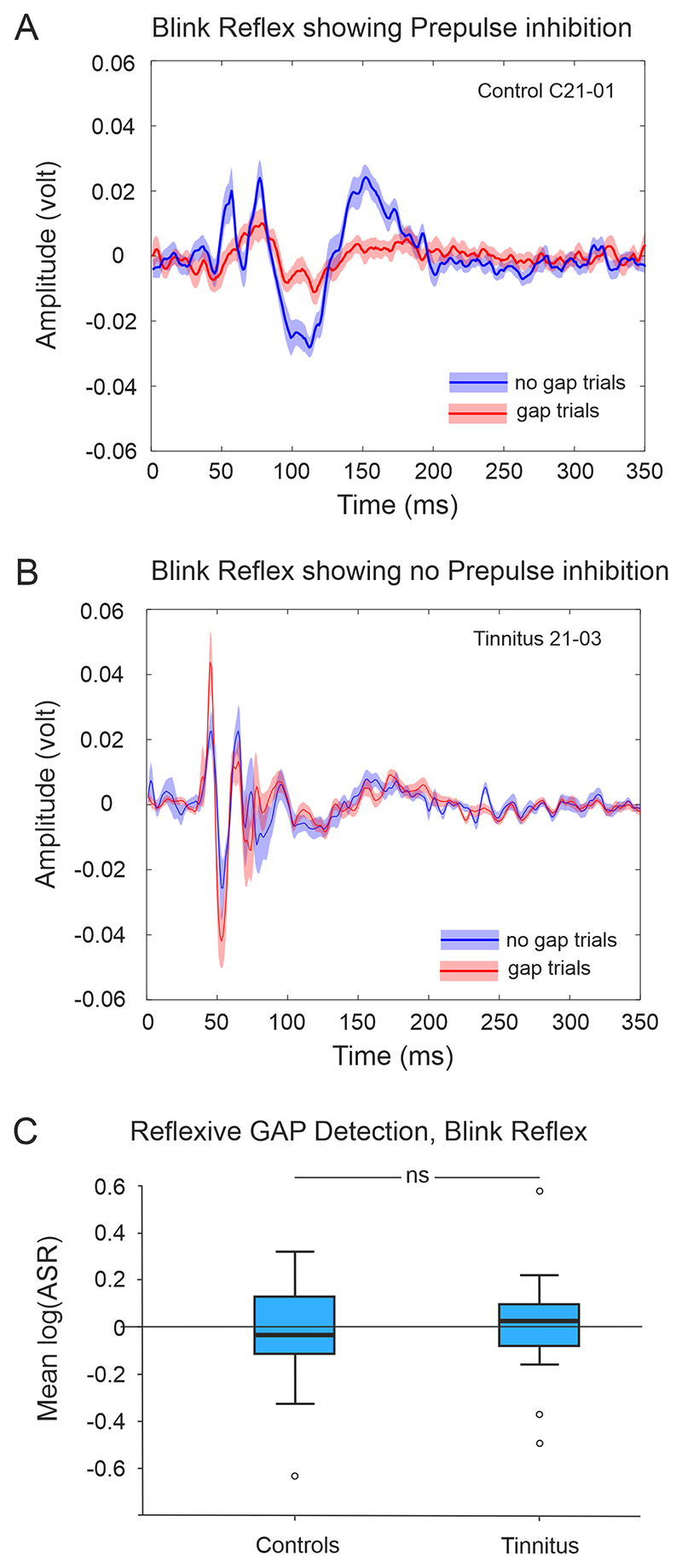
Exemplars of the recorded blink reflex in response to loud BBN presented in noise and preceded (gap trials) or not by a silent gap (no gap trials). (A) Control subject showing prepulse inhibition and (B) for a tinnitus participant who does not show prepulse inhibition. Lines represent the mean across trials and shadowed areas the standard error of the mean. (C) Comparison of bootstrapped means (log(ASR) showing no differences between control (n = 27) and tinnitus (n = 12) groups (*p >* 0.05, *t*-test)). ASR, auditory startle response; ns, no significance.

We assessed control and tinnitus participants’ eye blink reflex using surface electrodes to detect the contraction of their orbicularis oculi muscle in response to a startle stimulus (Fig. 8A,B). Participants for which blink responses were not recorded in >50% of trials were categorised as blink-fails and were excluded. Blink-fails occurred because of early habituation or a low signal-to-noise ratio that could be observed at any time during testing. The blink-fail rates for control and tinnitus groups were 26% and 27% respectively. Participants categorized as non-blink-fails and included in the analysis rarely exhibited habituation across the time course of the testing session. Although some participants from either the control or tinnitus groups presented a very stereotyped blink responses across trials, others had a much greater variability across trials. Therefore, instead of simply calculating the mean over all trials, we bootstrapped log(ASR) with 2000 samples (ASR = a vector of all possible combinations of gap and no-gap trial amplitude ratios) to generate a distribution of means for each participant. We then compared the means of each distribution. Over 50% of control participants showed pre-pulse inhibition (Fig. 8). We found no difference in mean log(ASR) between control and tinnitus groups (*p >* 0.05, *t*-test. Fig. 8). There was no significant difference in the distributions of mean log(ASR) between tinnitus subgroups classified according to CTI (*p* = 0.06, Kruskal-Wallis). There was no significant difference in the prepulse inhibition between controls and tinnitus. However, this result is difficult to interpret since very few participants showed a consistent blink response. For example, 50% of control participants did no show prepulse inhibition regardless of the gap length.

Blink response typically consisted of several peaks (Fig. 8) that lasted around 150–200 ms. Latency of the first peak was around 50 ms. We analyzed the response latencies from gap and no-gap trials between control and tinnitus groups. We calculated an average response latency to gap and to no gap trials. All groups were normally distributed, so we conducted a one-way ANOVA, and found no significant difference between control and tinnitus groups for either gap or no gap trials (gap trials *F*_(1,41)_ = 1.867, *p* = 0.179; no gap trials *F*_(1,41)_ = 0.331, *p* = 0.959).

### 3.6 ABRs Revealed Some Differences between the Tinnitus Subgroups

**Fig. 9:**
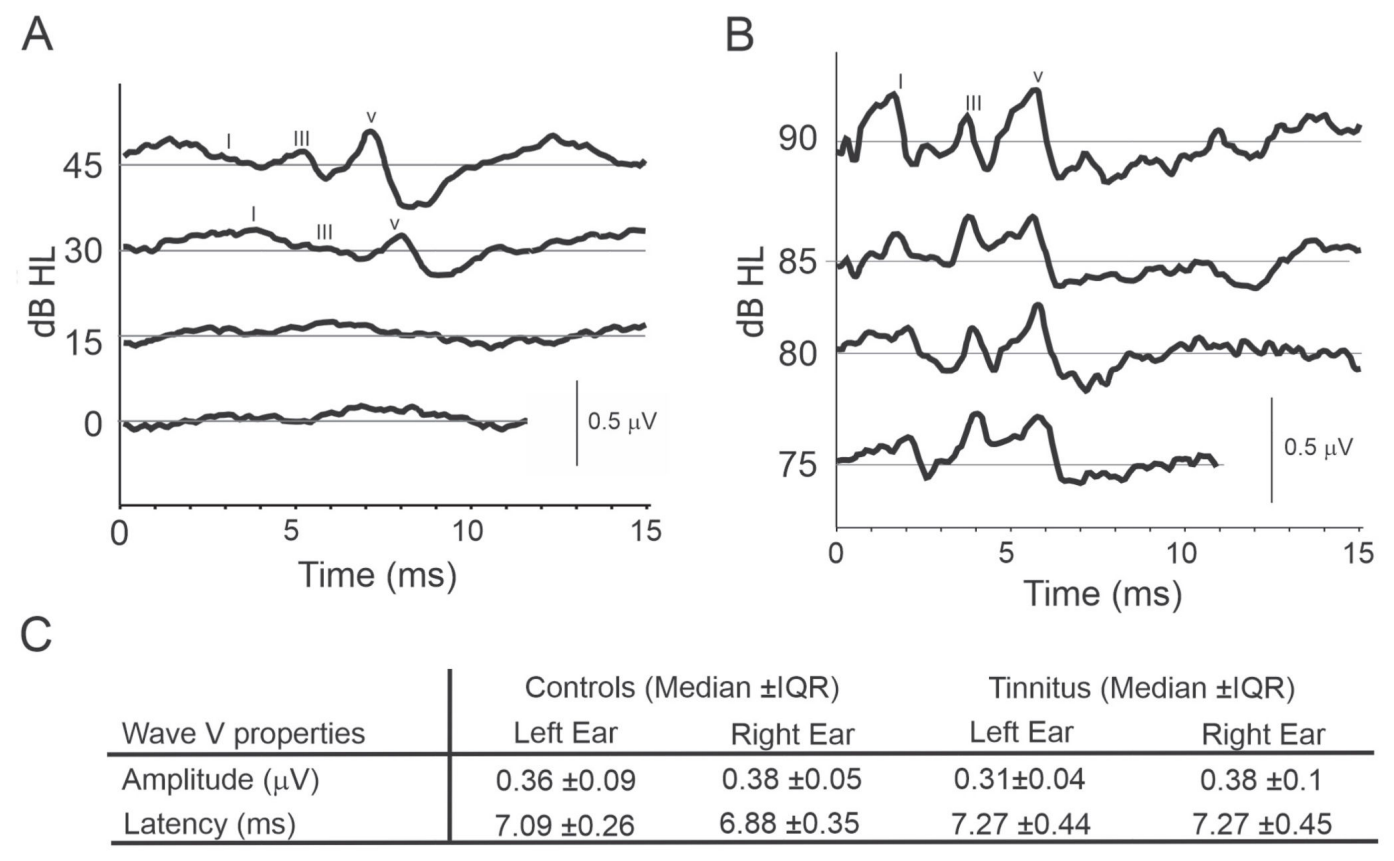
Auditory Brainstem Responses (ABRs). (A) Exemplary ABRs for low intensity hearing level clicks presented on the left ear, participant (2021C01). (B) Exemplary ABRs for high intensity hearing level clicks presented on the right ear, participant (2022C16). (C) Wave V amplitudes and latencies for the left and right ears. *Post hoc* analysis demonstrated that there were no significant differences between control (n = 16) and tinnitus (n = 12) groups (median ± interquartile range (IQR). Mann-Whitney *U* -test, *p >* 0.05). dB HL, decibels hearing level.

**Fig. 10:**
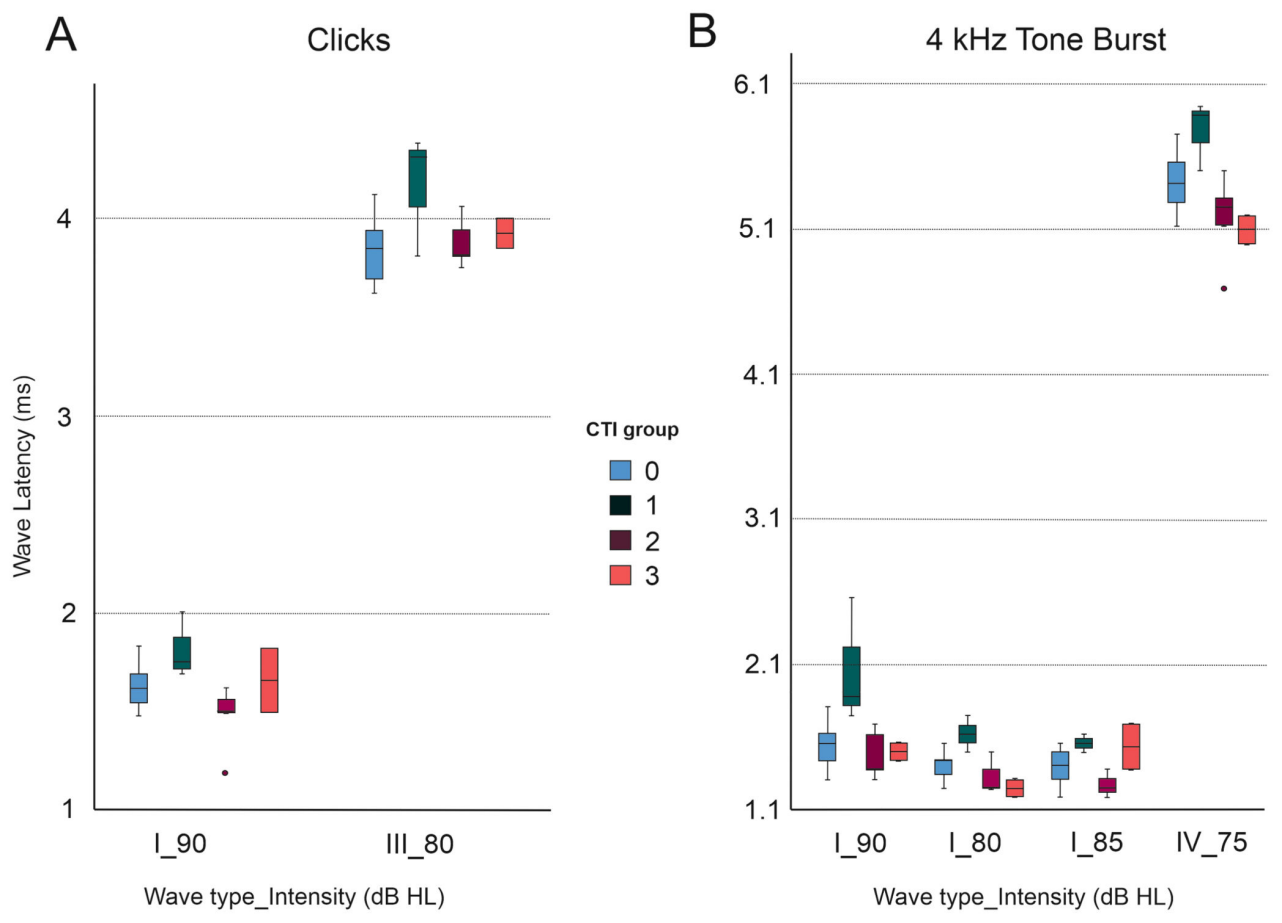
Latencies of different ABR waves (I, III, and IV) according to stimulus type. There were statistically significant differences between tinnitus subgroups classified according to CTI grade. CTI 1 had an elevated mean wave latency for waves I and III at 90 Hz and 80 Hz intensities, respectively (A). This difference in the CTI 1 group compared to other tinnitus CTI subgroups was also observed for 4 kHz tone bursts stimuli (B) (One-way ANOVAs and *post-hoc* Bonferroni multiple comparison analysis, with an adjusted *p*-value of *p* = 0.0083).

ABRs were recoded with Sentiero equipment using clicks and pure tone bursts (2 and 4 kHz tone burst (TB)) presented through headphones. Initially, we used 0, 15, 30, and 45 dB HL (Fig. 9). However, the peak ABR wave amplitudes were too low, especially wave I, so in subsequent participants we used 75, 80, 85, and 90 dB HL (Figs. 9,10).

ABR traces were similar across all three stimulus types (Clicks, 2, and 4 kHz TB). Typical traces can be found in Fig. 9. Three waves (I, III and V) were identifiable in most participants, with waves becoming more obvious from I to III to V. All stimulus types elicited a predictable increase in wave latency as stimulus intensity decreased. Invalidity rates for each stimulus type, when the trace was not good enough to extract data, were similar overall, at 10.7%, 8.3% and 7.4% for 2 kHz, 4 kHz TB and Click stimuli respectively.

The amplitude (Pearson’s correlation coefficient, *r* = 0.77, *p <* 0.01) and latency (Pearson’s correlation coefficient, *r* = 0.59, *p <* 0.05) of wave V responses to clicks between the left and right ear were well correlated in the control group. However, the amplitude (Pearson’s correlation coefficient, *r* = 0.05, *p* = 0.86) and latency (Pearson’s correlation coefficient, *r* = 0.66, *p* = 0.40) were not well correlated between ears in the tinnitus group. This lack of correlation may be indicative of tinnitus and/or a hidden hearing loss associated with tinnitus. Alternatively, it could be because of the limited data available from tinnitus participants.

The fact that a lower proportion of tinnitus participants elicited detectable responses (33%) than in the control group (62%) may be important, but this difference was not significant (Chi-squared test, *χ*^2^ = 1.7, *p* = 0.19). There was no significant difference in the amplitude or latency of wave V responses between the control and tinnitus groups when testing the left or right ears (Mann-Whitney *U* -test, *p >* 0.05 for each test).

Statistical testing revealed no significant differences in the wave latency of either wave I, III or V, for either click, 2 kHz TB or 4 kHz stimuli, at any intensity, between control and tinnitus groups (Fig. 10 and Table 6). However, significant differences were found between the tinnitus cohort when stratified by CTI grade (Fig. 10 and Table 6). CTI 1 group (slight tinnitus) had an elevated mean wave latency for several wave types (I, III, and V) and intensities for click and 4 kHz TB stimuli, compared to other CTI groups (CTI2 and CTI3, mild and moderate tinnitus, respectively) (One-way ANOVAs and *post-hoc* Bonferroni multiple comparison analysis, with an adjusted *p*-value of *p* = 0.0083) (Fig. 10 and Table 6).

## 4 Discussion

Current methods that seek to quantify tinnitus severity, such as the standardised questionnaires used in this study, focus solely on the subjective measures of experience and perceived impact of tinnitus on daily living. However, given the frequent association of tinnitus with explicit and/or hidden hearing loss, such subjective measures could in future be complemented by objective techniques that quantify any underlying hearing loss (including the magnitude and etiology) and associated behavioral effects. Whilst standard audiometry and ABRs are capable of measuring hearing loss, they fall short of capturing its downstream behavioral effects [4–6]. The behavioral effects of tinnitus can be observed in two domains. Within the auditory domain, tinnitus is thought to manifest as decreased speech intelligibility, due to either impaired temporal resolution or increased listening effort because of impaired sound-in-noise filtering ability. Outside the auditory domain, it can manifest as sleep disturbances and depression (in the most severe cases) [1–3].

We aimed to improve the operant silent gap detection paradigm used in animal models of tinnitus [13,17,19,36, 37] for the identification of tinnitus in humans. Additionally, we aimed to explore the paradigm’s potential use in clinical and experimental contexts to improve the treatment and aetiological understanding of tinnitus. One of the main issues with using the operant silent gap detection paradigm in humans is that humans have a significantly higher temporal sensitivity than animal models [38]. This results in a ceiling effect, whereby maximum performance is attained at much shorter gap durations. This ceiling effect reduces discriminability. In the present study, we have explored altering several variables other than the duration of the gap, to overcome this ceiling effect. Varying the predictability and depth modulation of the silent gap resulted in reduced performance from that previously reported [38]. Using depth modulated gaps together with stimuli of different spectral content, we were able to discriminate between control and tinnitus groups at the population level.

Using performance in the gap detection paradigm as a proxy for temporal resolution across the frequency range has already been described in animal models and humans with normal hearing. In both cases, animals and humans performed better at discriminating silent gaps for higher frequency sounds [36,38–42]. Previous studies using animal models of hearing loss [40] and tinnitus [20] have used the gap detection paradigm to make it more suitable for tinnitus identification [13]. In this study, we have explored the use of a gap detection paradigm in humans to discriminate both between tinnitus and control groups, and between tinnitus participants grouped by tinnitus severity. We have shown that tinnitus participants demonstrate the same trend as controls in the variation of temporal sensitivity across the frequency range. Nevertheless, tinnitus participants did have an overall deficit in gap detection. This might suggest that temporal resolution is affected across the hearing range, not just close to their tinnitus frequency. However, when we analysed performance for the various stimulus types in each frequency band used, greater differences were found for the NBN centered at 8 kHz (side frequencies 5.6 and 11.3 kHz). This approximately corresponds to the frequency of the tinnitus percept experienced by most of our tinnitus participants (left 9.23 and right 8.97 kHz, Table 3). This relationship is predicted by the frequency edge effect model [43]. In addition to the differences found at 8 kHz, close to the matched tinnitus frequency, we also found greater differences at 2 kHz. This frequency region is the most sensitive area in the human audiogram and where gap detection performance in control participants is better (Fig. 5). Therefore, it is possible that the resolution of the gap detection task is higher at this frequency, and consequently that 2 kHz stimuli are superior able to capture the differences between control and tinnitus groups.

The association between tinnitus pitch perception and audiometric sensitivity has already been demonstrated (for example [44–46]). This association predicts that the frequency of the tinnitus percept could lie within [44,47,48] or more often on the edge of [49,50] the frequency range of any hearing loss. This association could help discriminate between different tinnitus subtypes, as suggested by Vanneste and De Ridder [51]. These subtypes could be differentiated by the mechanism of hearing loss, such as the degeneration of cochlear nerve fibres (auditory neuropathy [7,52]) without changes in the audiogram thresholds, so called hidden hearing loss [4], age-related hearing loss [53], hearing loss induced by noise overexposure [54] and hearing loss caused by ototoxic drugs [55].

The results from our gap detection paradigm contradict an animal study using a salicylate-induced rat model of tinnitus [36] and two other human studies [22,56] that did not report changes in the gap detection with tinnitus. These discrepancies could be explained by the use of a non-clinically relevant model of chronic tinnitus induction (the salicylate model) in the animal study [36], testing with only 50 ms silent gaps that were too long and predictable in the first referenced human study [56], and the different characteristics of the participants with an associated audiometric hearing loss in the other referenced human study [22]. We suggest that our operant conditioned testing protocol maximized any differences between control and tinnitus participants.

Our GPIAS and ABR results show no differences between tinnitus and control participants although some differences were observed in the ABRs when tinnitus groups stratified by subjective tinnitus severity were compared (see Table 6 and Fig. 10). However, these differences should be viewed with caution due to a low sample size and variability recording quality. Differences in GPIAS between tinnitus and control human participants might be more evident under monaural rather than the binaural presentation [20,57]. Differences in ABRs, mainly with changes in the amplitude and latency of wave I, have also been reported between tinnitus and control participants [58–60]. Changes in ABRs depending on tinnitus duration have been described else-where [61,62] and it is encouraging that our data show that latency of wave V could be used to discriminate between tinnitus subtypes (Table 6). Therefore, ABRs could potentially be used to distinguish tinnitus, tinnitus subtypes and/or hidden hearing loss associated with tinnitus in the future.

Increasingly, researchers are accepting that tinnitus is a heterogenous condition [63]. Tinnitus duration and associated symptomatic distress seem to correlate well with activation of different brain areas beyond the auditory cortex [58]. In fact, TFI scores in this study revealed that, paradoxically, the CTI 1 group had subjectively higher auditory dysfunction than CTI 2 and CTI 3 (although not statistically significant) despite their tinnitus being less bothersome in all other domains. To fully establish whether CTI 1 represents a less bothersome but objectively/audiologically more severe tinnitus subtype, we need to recruit more participants with specifically chronic “non-bothersome” tinnitus. Although the present study intended to recruit tinnitus participants across all severity levels, maybe because of the relatively young age and lack of hearing loss criteria, the number of participants with more severe tinnitus was underrepresented. It will be necessary for future experiments to have more equal numbers of individuals in each tinnitus severity group.

The future holds great promise for the management of tinnitus with new treatment strategies aiming to target brain plasticity, including vagus nerve stimulation [64,65] or combining auditory and somatosensory stimulation [14,66]. Our research supports the notion that the settings we used in the operant silent gap-in-noise detection paradigm are optimized to reduce the ceiling effect seen in humans and maximize the difference between tinnitus and control participant scores. We offer a proof-of-concept to support the use of objective measurements for tinnitus in the assessment of future treatments. Although the gap-in-noise test in its current form is unable to distinguish individual tinnitus sufferers, it might be used to test the efficacy of therapeutic tools in the same tinnitus cohort before and after treatment.

## 5 Conclusions

Operant silent gap-in-noise detection is impaired in tinnitus participants when the experimental paradigm is adjusted to reduce the predictability and saliency of the silent gap. The behavioral paradigm can distinguish tinnitus and control groups suggesting that temporal resolution is impaired in tinnitus. However, in young adults with normal hearing, the paradigm is unable to objectively identify tinnitus at the individual level.

The differences between tinnitus and control groups found with operant testing could not be replicated by a reflexive behavioral paradigm. This suggests that, in humans, operant silent gap-in-noise detection is more sensitive than GPIAS to objectively identify individuals with tinnitus.

In addition, operant behavior and ABRs data show interesting differences across the tinnitus cohort when stratified by tinnitus severity. These require further confirmation by repeating with larger sample sizes and investigating individuals with more severe tinnitus (CTI 4 and 5).

## Supplementary Material

Supplementary material associated with this article can be found, in the online version, at https://doi.org/10.31083/j.jin2310183.

Supplementary Figs 1 & 2

## Figures and Tables

**Table 1:** Hit and lapse rates in the silent gap-in-noise paradigm according to gap position (mixed-effects ANOVA).

Position of gap	Hit rate					Lapse rate
	1 ms	2 ms	3 ms	4 ms	10 ms	
Beginning	0.25 ± 0.02	0.31 ± 0.02	0.34 ± 0.02	0.49 ± 0.02	0.83 ± 0.02	0.17 ± 0.02
Middle	0.28 ± 0.02	0.30 ± 0.02	0.40 ± 0.02	0.54 ± 0.02	0.87 ± 0.01	0.13 ± 0.01
End	0.26 ± 0.02	0.26 ± 0.02	0.32 ± 0.02	0.43 ± 0.02	0.78 ± 0.02	0.22 ± 0.02
Significance	*F*_(2,635)_ = 0.33 *p* = 0.72	F_(2,635)_ = 0.18 *p* = 0.16	F_(2,635)_ = 0.47 *p* = 0.01	F_(2,635)_ = 6.06 *p* < 0.01	F_(2,635)_ = 5.63 *p* < 0.01	F_(2,635)_ =5.63 *p* < 0.01

ANOVA, analysis of variance.

**Table 2:** Demographics of Control and Tinnitus Participants.

	Control Median (Quartiles)	Tinnitus Median (Quartiles)	Mann-Whitney U Test
Number Participants	33	20	
Age	20(20–21)	19.25 (20.5–21.75)	U = 855.5; *p* = 0.950
Gender ration (M/F)	0.83	1.2	U = 859.5; *p* = 0.696
University student ratio	0.89	0.94	U = 838.5; *p* = 0.829
Free school meals ration	0.06	0.05	U = 859.5; *p* = 1
Best musical grade	8 (6–8)	4(0.75–3.5)	U = 755; *p* = 0.0014**
Languages spoken	2 (1–2)	2 (1–2)	U = 847; *p* = 0.8154
Handedness *EHI*	88.23 (76.5–100)	83.3 (66.7–86.7)	U = 976; *p* = 0.0511
Hyperacusis *HQ*	25 (15–29)	39 (28.5–46)	U = 684.5; *p* = 0.0018**
Hearing handicap *HHIA*	0 (0-0)	0.06 (0.05-0.10)	U = 675.5; *p <* 0.001**

*EHI*, Edinburg Handedness Inventory; *HQ*, Modified Khalfa Hyperacusis Questionnaire; *HHAI*, Hearing Handicap Inventory Assessment. The music grade system in the UK for voice and musical instrument goes from Grade 1 to Grade 8. Grade 1 is at entry level and Grade 8 the typical standard required for entry to a Music College. ** indicates statistical significance *p <* 0.01.

**Table 3:** Characterization of the tinnitus percept.

	Tinnitus Left Ear Median (quartiles) Tinnitus		Right Ear Median (quartiles)
Frequency (kHz)	9.23 (4.25–13.21)		8.97 (4.56–13.35)
Intensity (dB SPL)	22(17–33.5)		26 (20–36.7)
TFI		14(12.8–24.6)	
THI		22(14.5–32)	
Tinnitus Duration (months)		44 (21–70)	

TFI, Tinnitus Functional Index; THI, Tinnitus Handicap Inventory; SPL, sound pressure level.

**Table 4:** Gap detection threshold at 65% hit rates (65% GDT) and inflection point (ipGDT) for each type of stimulus used.

Frequency (Hz)	Control	65% GDT	ipGDT
1 NBN	Mean	7.25	4.74
	Median	5.99	4.24
	Variance	27.21	12.69
	Spearman’s Correlation	0.94 (*p* < 0.001)	
2 NBN	Mean	7.10	5.88
	Median	6.20	5.40
	Variance	17.38	10.38
	Spearman’s Correlation	0.97 (*p* < 0.001)	
4 NBN	Mean	4.87	3.77
	Median	4.58	3.80
	Variance	3.91	2.36
	Spearman’s Correlation	0.82 (*p* < 0.001)	
8 NBN	Mean	3.66	2.82
	Median	3.64	3.11
	Variance	1.78	1.18
	Spearman’s Correlation	0.86 (*p* < 0.001)	
16 NBN	Mean	3.75	2.71
	Median	3.35	2.59
	Variance	6.82	5.21
	Spearman’s Correlation	0.87 (*p* < 0.001)	
BBN	Mean	3.13	0.45
	Median	1.56	0.17
	Variance	9.85	0.30
	Spearman’s Correlation (NA because 65% GDT BBN had a sample size <3)		

BBN, broadband noise and one octave narrowband noise centred at 1, 2, 4, 8, and 16 kHz. NA, not applicable.

**Table 5:** Tinnitus functional index (TFI) and tinnitus handicap inventory (THI).

TFI score	Tinnitus cohort	CTI 1	CTI 2	CTI 3
	Median (quartiles)	Median (quartiles)	Median (quartiles)	Median (quartiles)
Overall	14.4(12.8–31.4)	12.8(6.2–14.1)	20(15.2–31.4)	40.8 (39.2–46.4)
Intrusive	0.25 (0.17–0.33)	22.5 (20–29.7)	23.3 (16.7–31.7)	43.3 (33.3–63.3)
Sense of control	25 (17.5–33.3)	28.3 (3.33–35.8)	46.7 (24.7–58.3)	60 (50–73.3)
Cognitive	38.3 (16.7–55)	5 (0–6.67)	20 (10–48.3)	56.7 (43.3–56.7)
Sleep	10 (3.33–40)	3.33 (0–12.5)	10 (4.3–30)	40 (40–96.7)
Auditory	6.67 (11.7–43.3)	8.3 (3.33–27.5)	6.67 (0–10)	3.33 (0–6.7)
Relaxation	20 (13.3–49.2)	13.3 (7.5–20)	23.3 (15–58.3)	56.7 (50–66.7)
Quality of life	6.25 (2.5–11.9)	2.5 (0–4.40)	10 (6.25–11.3)	25 (17.5–30)
Emotional	11.7(3.33–22.5)	5 (0–10)	16.7 (6.67–31.7)	43.3 (30–43.3)
THI score	22 (14–24)			

CTI, combined tinnitus index.

**Table 6:** Auditory Brainstem Evoked responses (ABRs).

Stimulus type	Wave type	Intensity (dB SPL)	ANOVA (between group comparison)	Bonferroni (pairwise comparison)
Click	I	90	*p* = 0.011	CTI 1>CTI 2, *p* = 0.008**
Click	III	80	*p* = 0.046	CTI 1>CTI 0, *p* = 0.036*
4 kHz TB	I	85	*p* = 0.016	CTI 1>CTI 2, *p* = 0.023*
4 kHz TB	I	80	*p* = 0.001	CTI 1>CTI 0, *p* = 0.011*
				CTI 1>CRI 2, *p* = 0.003**
				CTI 1>CTI3, *p* = 0.003**
4 kHz TB	I	75	*p* = 0.002	CTI 1>CTI 0, *p* = 0.002**
				CTI 1>CTI 2, *p* = 0.003**
				CTI 1>CTI 3, *p* = 0.028*
4 kHz TB	V	75	*p* = 0 002	CTI 1>CTI 2, *p* = 0.004**
				CTI 1>CTI 3, *p* = 0.010*

TB, tone burst. Statistical significance * *p <* 0.05, ** *p <* 0.01.

## Data Availability

Data will be shared upon requests.
