# Supplementary material for: Optimization of the Operant Silent Gap-in-Noise Detection Paradigm in Humans
Source: J Integr Neurosci. Author manuscript; Available in PMC 2025 Jul 29. (PMC7617955; doi:10.31083/j.jin2310183)
Supplement: Supplementary Figs 1 & 2 [file EMS207103-supplement-Supplementary_Figs_1___2.docx]

**Supplementary Figure 1.** **Response times on the gap detection task.** (**A)**, mean ±SE response times for different gap duration trials across all participants (n=18) and all stimulus types. (**B)**, mean ±SE response times for different stimulus types trials across all participants (n=18) and all gap duration trials.

**Supplementary Figure 2.** **Correlation between Gap detection thresholds and tinnitus suffering duration** (**A**), Threshods at 65% performance. Spearman’s Coefficient (rho); *p-*value NBN 1kHz 0.683, *p*= 0.007; NBN 2kHz 0.691, *p*= 0.004; NBN 4kHz 0.645, *p*= 0.013; NBN 8kHz 0.331, *p*=0.27; NBN 16kHz 0.116, *p*=0.72 (**B**) Thresholds at 65% performance. NBN 1kHz 0.506, *p*= 0.054; NBN 2kHz 0.644, *p*= 0.01; NBN 4kHz 0.747, *p*= 0.002; NBN 8kHz 0.309, *p*=0.28; NBN 16kHz -0.026, *p*=0.93; BBN 0.107; *p*=0.68.
